# The COPII Transport Complex Participates in HPV16 Infection

**DOI:** 10.3390/v17050616

**Published:** 2025-04-25

**Authors:** Patricia M. Day, Cynthia D. Thompson, Andrea S. Weisberg, John T. Schiller

**Affiliations:** 1 Laboratory of Cellular Oncology, NCI, NIH, Bethesda, MD 20892, USA; thompscy@mail.nih.gov (C.D.T.); schillej@dc37a.nci.nih.gov (J.T.S.); 2 Laboratory of Viral Diseases, NIAID, NIH, Bethesda, MD 20892, USA

**Keywords:** HPV, papillomavirus, pseudovirus, trafficking, mitosis, Golgi, COPII, ER, ERES

## Abstract

Human papillomavirus (HPV) 16 is transported in a retrograde fashion from the cell surface to the Golgi apparatus. Prior to mitosis, the virus loses association with the Golgi and, following nuclear envelope breakdown, is found associated with the condensed mitotic chromatin. The intervening steps have not been well defined. It was previously demonstrated that the virus is transported to the mitotic chromosomes in vesicles. Here, we describe the role of the endoplasmic reticulum (ER) in the post-Golgi trafficking and the importance of the ER-generated coat protein complex II (COPII) anterograde trafficking pathway in HPV infection. HPV pseudovirus (PsV) colocalized with COPII components and silencing of this pathway inhibited HPV infection. Additionally, the inner COPII coat protein, Sec24b, could be biochemically isolated in association with HPV capsid proteins. This study provides insight into the mechanism of post-Golgi HPV trafficking.

## 1. Introduction

Human papillomaviruses (HPVs) comprise a large family of nonenveloped DNA viruses that include a number of important human pathogens. The high-risk subset of HPVs, of which HPV16 is the most prominent, include the causative agents of multiple cancers including almost all cases of cervical cancer (reviewed in [1]). Apart from its medical importance, the investigation of HPV entry and intracellular trafficking pathways has yielded information applicable to general cell biological studies. The HPV infectious pathway has emerged as an anomaly within the currently described spectrum of viral endocytic routes. HPV virions have an exceptionally asynchronous infectious process, with a protracted average cell surface residence time [2,3,4,5]. They are endocytosed by a non-clathrin, non-caveolar pathway most closely resembling macropinocytosis [2]. The particles proceed in a retrograde vesicular pathway from the endosomal compartment to the Golgi apparatus [3,4]. Following transit through the Golgi, the virus emerges, essentially intact, in vesicles that associate with the host cell DNA during open mitosis [5,6,7,8]. The virus hitchhikes on the mitotic chromatin for passage into the nucleus following nuclear envelope reformation. Intranuclear dissolution of these vesicles and the viral capsid then follows [3]. This strategy is unique among known viral entry schemes. In this study, we have more closely examined the passage of the virions from the Golgi to the mitotic chromosomes.

The 50–60 nm PV capsid is composed of two proteins, the major capsid protein L1 and the minor capsid protein L2. L1 can self-assemble into virus-like particles (VLPs), which are antigenically indistinguishable from the authentic virion (reviewed in [4]). Despite its minor structural role, L2 is the mediator of multiple essential steps during the intracellular trafficking process (reviewed in [5]). L2, initially largely buried within the capsid shell, is progressively exposed as entry progresses. It is processed by furin [6] prior to endocytosis and, following entry into early endosomes, is partially extruded into the cytoplasm in a gamma secretase-dependent manner [7]. This exposes L2 regions that sequentially interact with cytoplasmic cellular proteins that provide essential guidance of the capsid-containing vesicles to the Golgi. These host components include the retromer and commander complexes, sorting nexin 17 (SNX17) and coat protein complex I (COPI) coat proteins among others [8,9,10,11,12]. The L1 capsid structure remains luminal during this passage [13]. HPV pseudoviruses (PsV) were used for these critical studies, in addition to the majority of HPV entry and infection research since their development in 2004 [14]. The PsV production method takes advantage of the propensity of the capsid proteins to self-assemble around a marker plasmid, which serves as a pseudogenome to allow quantification of successful infection.

It is well established that entry into mitosis and the accompanying nuclear envelope breakdown are essential for the successful expression of the HPV pseudogenome [15,16,17]. As the Golgi is transformed from an organized, directional stack into small, dispersed vesicles during mitosis (reviewed in [18]), it has been suggested that this is the source of the virus-laden vesicles observed [13]. However, in addition to the well-described Golgi localization, HPV L2 and the pseudogenome have also been localized to the endoplasmic reticulum (ER) [19,20,21]. This observation has not been as fully explored, and the role of transit through the ER has not been mechanistically connected to the described entry pathway.

We decided to examine the ER as a potential source of the vesicles involved in the transit of the capsid to the mitotic chromosomes. This study was motivated in part by the observation of the Campos laboratory that early in mitosis, prior to chromosomal association, PsV dramatically lost colocalization with Golgi markers [17], and by the previous description by the Meneses laboratory that STX18, an ER protein that can act in concert with ER exit pathways, plays a role in PV infection [8,22]. As an additional impetus, we had previously visualized, by transmission electron microscopy (TEM), the morphological characteristics of the chromosome-associated vesicles containing HPV16 PsV. Interestingly, many capsids were found within extended vesicles containing multiple particles [23]. This is not typical of either Golgi-derived transport vesicles or Golgi-derived mitotic vesicles [24,25]. COPI-coated vesicles, which mediate the retrograde transport through the Golgi and into the ER, have been demonstrated to have an average inner membrane diameter of 50 nm [24]. The diameter of mitotic Golgi vesicles was estimated to be 50–57 nm [25]. However, COPII-coated vesicles, which mediate anterograde traffic from the ER, specifically the ER exit sites (ERES) to the Golgi, range in size from 60 nm vesicles to 200 nm tubules after detachment from the ER [26,27,28]. Therefore, we examined the possibility that infectious entry of HPV includes a transit step through the ER and that export from the ER defines the penultimate step prior to chromosome association and ultimately nuclear entry.

## 2. Materials and Methods

### 2.1. Pseudovirus and VLP Production

PsV preparations were produced according to the protocol published on the laboratory website (https://ccrod.cancer.gov/confluence/display/LCOTF/Home, accessed 15 March 2022). The protocol for production of fully mature standard PsV was utilized followed by ultracentrifugation through an Optiprep (iodixanol) step gradient. Briefly, 293TT cells were transfected with a bicistronic plasmid, p16SheLl encoding the HPV16 L1 and L2 proteins together with a reporter gene plasmid. The packaged marker plasmid varied according to usage: the non-fluorescent pYSEAP plasmid was packaged for microscopy-based experiments, and pfwB, encoding GFP, was for assessment of infection by flow cytometry. For immune-isolation of L2-associated proteins PsV containing HA-tagged L2 was produced using the p16Ll2-laugh plasmid. This bicistronic plasmid encodes an L2 protein fused with five carboxy terminus HA tags. SDS-PAGE analysis was performed to determine the concentration of the HPV16 L1 content of the pseudovirus preparation.

### 2.2. Immunofluorescence

HeLa cells (obtained from ATCC #CCL-2) were seeded onto 12 mm high precision glass coverslips (ThorLabs) placed in 24-well plates at a density of 7 × 10^4^/well. Cells were cultured overnight and infected with 30 ng viral particles per well and incubated for 24 h. Cells were fixed with 2% paraformaldehyde for 20 min at room temperature, washed three times with 200 mM glycine in PBS, and processed for immunostaining. Briefly, cells were permeabilized with 0.5% Triton ×100 in PBS for 5 min at room temperature, blocked for 20 min in 5% normal donkey serum in PBS and incubated with primary antibodies diluted in 2.5% normal donkey serum. All antibodies and appropriate dilutions are listed below. Following this incubation and washing, appropriate secondary antibodies, as indicated, were applied. Antibody dilution buffer was supplemented with 0.1% Brij58 to decrease surface tension, thereby reducing the required antibody volume. Following final washes, cells were incubated with 200 ng/mL DAPI solution for 5 min, and then coverslips were removed from the plate and inverted onto Prolong Glass Antifade Mountant (Invitrogen) on a glass slide.

Images were either acquired with a 63× objective on a Zeiss 780 confocal system interfaced with a Zeiss Axiovert 100M microscope (Jena, Germany) or a 63× objective on a Leica Stellaris 8 FLIM system interfaced with an inverted Leica DMi8 microscope (Wetzlar, Germany). Images were collated with Adobe Photoshop software (https://www.adobe.com/), and adjustments were applied consistently across experimental groups. The colocalization experiments for control infected cells were performed in six independent experiments. Colocalization was determined with the Leica Application Suite X software (version 5.3.0) throughout the regions selected as described for each figure. Resident perinuclear proteins defined that region and colocalization within the area determined through central Z-stacks. Peripheral regions were defined as areas distal to the nucleus and perinculear marker protein. The analyzed Z-stack was selected based on the focal plane for these distal vesicles.

### 2.3. HPV PsV Infection

To evaluate PsV infection efficiency, HeLa cells were seeded at a density of 7 × 10^3^/well in a 96-well plate. Following adherence, HPV16 PsV containing the packaged plasmid, pfwB, was added to the cells in a dilution series in triplicate. The percentage of GFP-transduced cells was determined by flow cytometric analysis at 48 h following infection. Cells were trypsinized and resuspended in PBS containing 2% FBS and 0.1% sodium azide, and plates were analyzed using a high throughput sampler on a Becton Dickinson FACSCanto II flow cytometer. Triplicate wells from each condition were also used to determine DNA content as detailed below. The universal siRNA control infection relative to mock-transfected cells was considered to be 100% infection. Statistical analysis was performed with GraphPad Prism Software (version 9.3.1), in which a one-tailed unpaired *t*-test was used to determine *p* values.

### 2.4. Cell Cycle Analysis

For determination of DNA content, trypsinized cells were pelleted by centrifugation and fixed in 70% cold ethanol for 30 min. Cells were washed in PBS twice and resuspended in PBS containing 20 μg/mL RNase and 40 μg/mL propidium iodide. Single cells, based on forward and side scatter profiles, were analyzed.

### 2.5. siRNA Transfection

Six-well plates were seeded with HeLa cells at a density of 4 × 10^5^ per well. Following adherence, cells were transfected with 10 nM total of siRNA, either individual or pools, using Dharmafect 1 transfection reagent according to the manufacturer’s directions. At 48 h, post-transfection cells were trypsinized and replated for three parallel analyses: 96-well plates for PsV infectivity assays, coverslips for microscopic analysis, and 6-well plates for Western blot analysis. Infectivity analyses were performed in triplicate with a minimum of six replicate experiments. Confirmation of protein knockdown was confirmed by Western blot for each experiment. Colocalization analyses by immunofluorescence was performed for a minimum of three experiments with combined results used for statistical determination.

All siRNA sequences used are listed in Table 1.

### 2.6. Western Blot Analysis

Cells were harvested by trypsinization and lysed at a density of 2 × 10^6^/mL in NuPAGE LDS sample buffer with NuPAGE sample reducing agent. Lysates were homogenized by spinning through QIAshredder columns (Qiagen, Oberkochen, Germany). Proteins were separated by SDS-PAGE using 4–12% Bis-Tris NuPAGE gels (Invitrogen) and transferred to PVDF membranes. The membranes were then processed for Western blot analysis. Primary and secondary antibodies were diluted in Tris-buffered saline (TBS) supplemented with 2 mM EDTA, 0.1% NP40, and 3% bovine serum albumin subsequent to blocking in the same buffer. Binding of the HRP-conjugated secondary antibody was detected with Western Lightning Plus ECL reagent (Perkin Elmer, Waltham, MA, USA) using an Amersham Imager 680 analyzer (GE Healthcare, Marlborough, MA, USA).

### 2.7. Electron Microscopy

All TEM images were acquired during our previous analysis of HPV nuclear entry, although none of these images was previously published [23]. The methods were described in that manuscript. Briefly, HeLa cells that were infected with HPV16 PsV for 24 h were fixed with 2% glutaraldehyde in 0.1 M cacodylate buffer (pH 7.4) and processed according to published techniques [29]. Micrographs were obtained with a Tecnai G2 Spirit transmission electron microscope (FEI/Thermo Fisher Scientific, Hillsboro, OR, USA) fitted with a Gatan Orius CCD camera (Gatan, Inc. Warrendale, PA, USA).

### 2.8. Crosslinking and Immunoprecipitation

HeLa cells were plated at a density of 6 × 10^6^ per 150 mM dish. Five plates were prepared for each of the four experimental conditions. The following day three conditions were infected with 15 μg of HPV16L1-L2 laugh PsV. At 6 h post infection, one infected group was supplemented with aphidicolin to a final concentration of 5 μg/mL, and NH_4_Cl was added to a second infected group to a final concentration of 20 mM. At 24 h post infection, the cells were rinsed with PBS and crosslinked with Dithiobis (succinimidyl propionate) (DSP) (Thermo). A 200 mM DSP solution was freshly prepared in DMSO and diluted to 2 mM in PBS. A total of 10 mL of this solution was added to each plate. Crosslinking was performed with gentle rocking for 60 min at room temperature. At this point the crosslinking solution was removed and the plates rinsed once with PBS. Then, 10 mL of quenching solution (20 mM Tris-HCl pH 7.4) was applied to each plate and incubated with rocking for 15 min. The quench was removed, and cells were rinsed twice with PBS. Following this, cells were scraped into 10 mL of cold PBS using a rubber policeman, and identical conditions were pooled. Cells were pelleted for 10 min at 900× *g*. The pellets were resuspended in 5 mL of IP/lysis buffer (1% Tx100, 150 mM NaCl, 50 mM Tris-HCl pH 7.5, 50 mM MgCl_2_) containing a protease inhibitor cocktail (Sigma) and held on ice for 30 min. Debris was removed by centrifugation for 20 min at 16,000× *g*, and the supernatant was subjected to immunoprecipitation. Immunoprecipitation was performed in a 15 mL low-binding tube at 4 °C overnight using the HA tag CUTANA antibody (EpiCypher, Durham, NC, USA). Following overnight incubation, antibody-bound proteins were isolated with Protein G Dynabeads (Invitrogen, Carlsbad, CA, USA) according to the manufacturer’s directions. Eluted proteins were subject to SDS-PAGE and Western blot analysis as detailed above. The data shown are representative of three independent experiments.

The antibodies used throughout this study are listed in Table 2.

## 3. Results

### 3.1. TEM Reveals Extended PsV-Containing Vesicles

**Figure 1:**
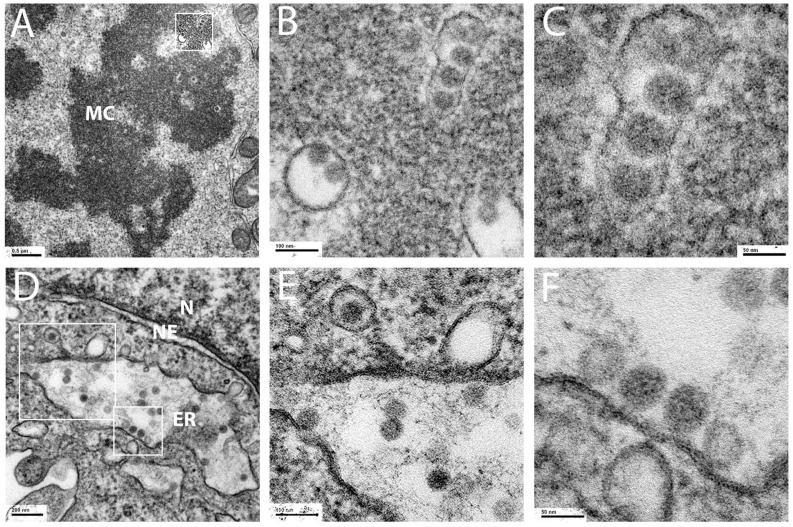
TEM micrographs of HPV16 PsV-infected HeLa cells. Cells were infected for 24 h with HPV16 PsV and processed for TEM imaging. A mitotic cell is shown in panels (**A**–**C**), with the condensed mitotic chromatin labeled MC. The panel inset box in A shows the area enlarged in the following panels. An interphase cell is shown in panels (**D**–**F**). The nucleus (N), nuclear envelope (NE), and endoplasmic reticulum (ER) are labeled accordingly. Two regions indicated in panel (**D**) are enlarged in the following two panels. Size bars are shown within each panel.

We recently demonstrated that, contrary to previous assumptions, HPV pseudovirions remain largely intact during their trafficking into the nucleus [23]. We described the presence of membrane-bound vesicles that contain 50 nm particles consistent with HPV16 pseudovirions, visualized by TEM of infected HeLa cells. These vesicles were found associated with the mitotic chromosomes and, more rarely, within the nucleus. Figure 1A–C shows a representative micrograph of these vesicles associated with condensed chromatin from a mitotic HeLa cell. These vesicles were only shown to be present following infection with L2-containing PsV. L1-only particle-“infected” and mock-infected cells contained no chromosome-associated vesicles, consistent with the requirement for L2 for correct trafficking [23]. We frequently observed vesicles that contained multiple virions. An example of multiple virus-containing vesicles associated with mitotic chromatin is shown in Figure 1B, a magnified area from Figure 1A. In one instance, a round vesicle, approximately 150 nm in diameter, containing two virions, is obvious. Another vesicle contains four virions. This vesicle is more tubular and extends to a length of approximately 250 nm. This vesicle is shown at a higher magnification in Figure 1C. As previously mentioned, these vesicle dimensions are not consistent with what has been described for Golgi-derived COPI transport vesicles, which mediate the retrograde transport through the ER. However, COPII vesicle size is known to be more variable due to the cellular requirement to export bulky cargoes, e.g., collagens. In some instances, to achieve assembly of these extended COPII vesicles, the transport and Golgi organization 1 protein TANGO1 (MIA3) in concert with the t-SNARE STX18, is employed [30]. STX18 has already been implicated as playing a role in HPV entry [8,22]. We also examined the capsid localization within interphase cells and found capsids associated with structures consistent with the ER (Figure 1D). This panel shows the nuclear membrane and an adjacent organelle, resembling the ER, containing multiple capsids. The two boxed regions are shown at a higher magnification. In Figure 1E, a region containing an extending bud containing a single particle is shown. This image is remarkably reminiscent of the ER-budding profiles previously imaged by TEM [31]. A region with four particles aligned along the luminal face of the membrane is shown in Figure 1F. We found no evidence in any of the interphase cells examined of vesicles containing capsids abutted to the nuclear membrane.

### 3.2. HPV16 PsV Localize to ERES and COPII Vesicles

Bovine PV (BPV) L2 was previously shown to colocalize with calnexin [8,22], and HPV16 L2 and the pseudogenome were found to localize with both glucose-regulated protein 78 (GRP78) and protein disulfide isomerase (PDI) [20,21]. GRP78 and PDI are both luminal proteins found within the rough ER, also referred to as ER sheets, and calnexin is an integral ER sheet membrane protein [32,33,34]. However, we had previously failed to find clear colocalization of the HPV16 pseudogenome with calnexin [35]. Despite this, due to our interest in the ER as a potential source of viral transport vesicles, we decided to reanalyze the ER localization and possibly more precisely define the intra-ER localization. We specifically focused on the secretory compartment, where COPII anterograde transport vesicles are generated. This more confined region could also allow for easier detection of pseudovirions due to an increased local concentration. Additionally, we also chose to use an anti-L1 reagent, which could increase the sensitivity of HPV16 detection compared to detection of the lower density L2. We previously demonstrated that the anti-HPV16 antibody, H16.7E, can detect L1 throughout the trafficking pathway as well as on both mitotic chromosomes and within the nucleus [23].

**Figure 2:**
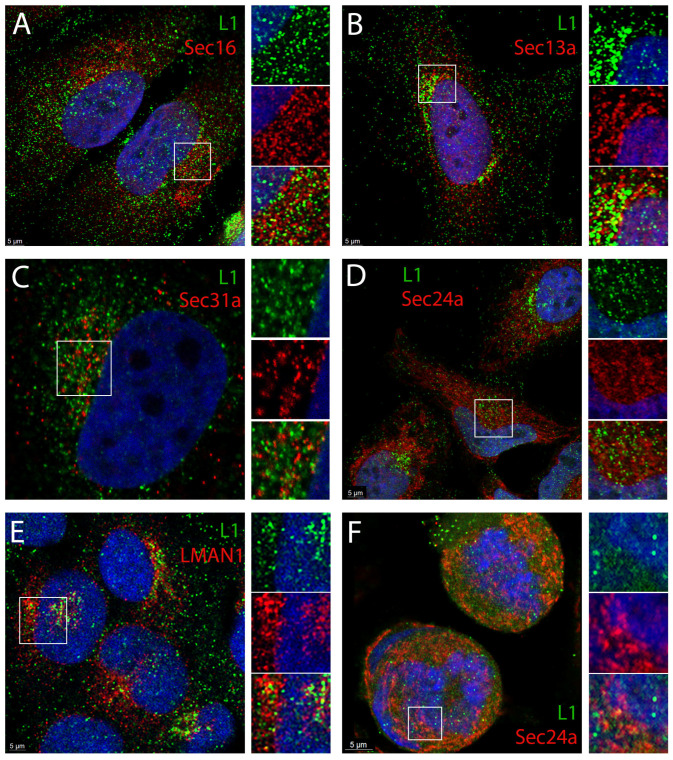
Colocalization of L1 with ERES and COPII marker proteins. HeLa cells were infected with HPV16 PsV for 24 h and processed for immunofluorescent staining. L1 was detected with the mouse antibody H16.7E in all panels. Co-staining with a panel of organelle marker proteins as indicated in each panel, is shown in panels (**A**–**E**). The localization of Sec 24a relative to L1 in prophase cells is shown in panel (**F**). An enlarged split channel image of the indicated area is shown to the right of each panel. All images are central sections from a z-stack collection. In each instance, L1 is displayed in the green channel and the cellular marker in the red channel. Nuclei are stained with DAPI (blue channel) in all panels. These analyses were confirmed in six independent experiments.

Sec16 is the defining protein of ER exit sites (ERES), where secretory cargo accumulates prior to anterograde transport [36,37]. This protein serves as the essential scaffold for the assembly of COPII vesicles around cargo [38]. We found clear colocalization of a subset of L1 (green) with Sec16 (red) as shown in Figure 2A. Split channels of the boxed area are shown on the right of the merged image. We also examined the localization of L1 relative to that of COPII vesicle components. COPII vesicle coats are comprise four cytosolic proteins that assemble into two concentric rings around their cargo (reviewed in [39]). The outer coat proteins, Sec13 and Sec31, encircle the inner coat protein complex Sec 23/24. The localization of L1 relative to Sec13a (red) is shown in Figure 2B. Again, the split channels of the selected region are shown on the right. Sec31a and anti-L1 staining is shown in Figure 2C, and L1 staining relative to Sec24a is shown in Figure 2D. The ER-Golgi intermediate compartment (ERGIC) is the recipient organelle of anterograde cargo from the ERES (reviewed in [40,41]). We examined the localization of L1 relative to LMAN1 (also known as ERGIC53), the characteristic marker protein of the ERGIC [42,43]. This staining is shown in Figure 2E. Thus, L1 colocalizes with both outer and inner COPII proteins within the ERES and likely traffics out of the ERES to the ERGIC. We did not expect more extensive colocalization than what is demonstrated as HPV PsV trafficking is asynchronous and would also be distributed throughout the retrograde pathway.

We next wanted to determine if we could detect the COPII coat proteins in association with L1 in mitotic cells. We examined the colocalization of L1 with Sec24a in prophase cells (Figure 2F). We could find colocalization in cytoplasmic regions immediately adjacent to the condensed chromatin. However, there is not strong colocalization detected within the chromatin. It is unclear if this is due to steric inaccessibility of the epitopes on coat proteins within the chromatin or the loss of the COPII coat from the L1-containing vesicle. Typically, it is thought that COPII coats are lost upon vesicle fusion with their target membrane (reviewed in [44]). However, in the described situation there is no target membrane to receive the vesicle cargo.

### 3.3. siRNA Depletion of COPII Transport Mediators Decreases HPV16 PsV Infection

**Figure 3:**
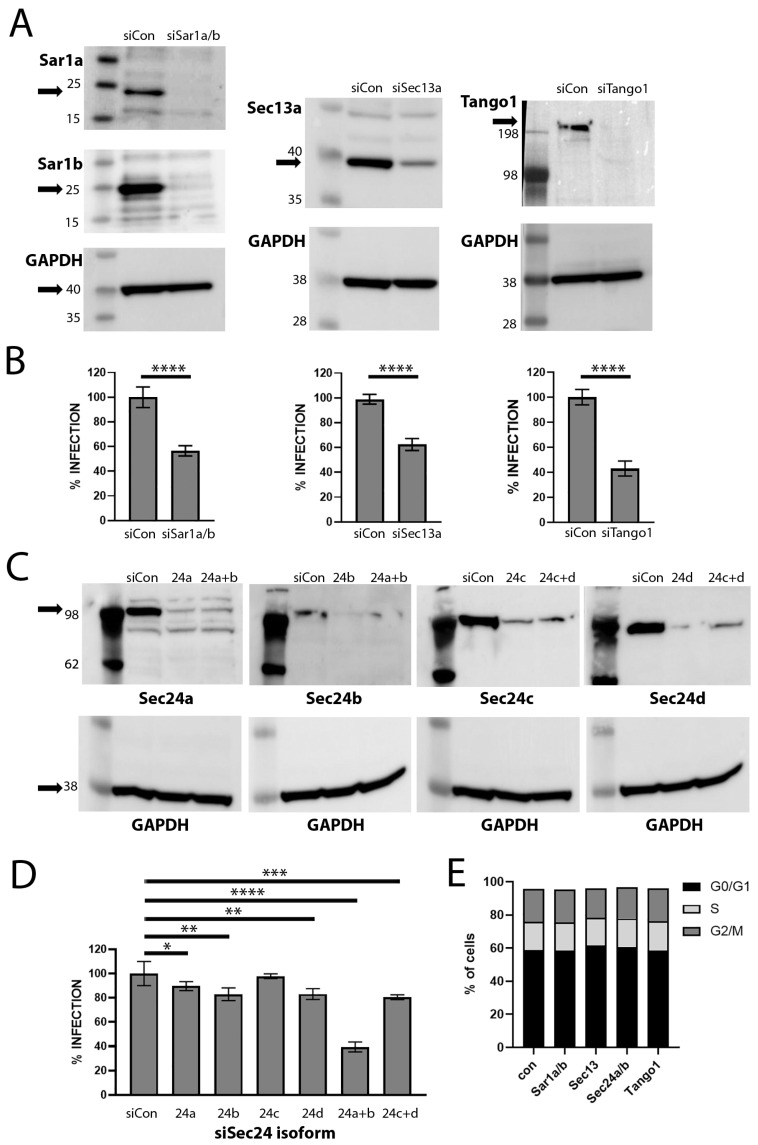
Silencing of COPII transport mediators affects HPV PsV infection. HeLa cells in 6-well dishes were transfected with the indicated siRNAs. At 48 h post transfection, cells were trypsinized and replated for PsV infection and Western blot analysis. Cells were lysed for Western blots at 72 h post siRNA transfection. Infection was evaluated at 48 h post virus addition, corresponding to 96 h post siRNA transfection. Panel (**A**) shows the protein expression for the indicated proteins under the conditions indicated above each lane. GAPDH was included as a loading standard and is shown in the lower panel in each instance. The expected protein migration is indicated by an arrow. The PsV infectivity, as determined by flow cytometry as percentage of GFP positive cells, is shown in panel (**B**). These results represent the compilation of multiple independent experiments. Additionally, each experiment was performed in triplicate. Sar1a/b depletion is the composite of nine experiments. The depletion of Sec13a was also replicated nine times. Tango1 depletion was performed in 12 separate experiments. Statistical significance < 0.0001 was reached in each instance (indicated by ****). Panel (**C**) shows the protein expression for the individual and combined siRNA transfection experiment for the Sec24 isoforms, as indicated. Panel (**D**) shows the infectivity analyses for these depletions. The only condition that was highly significant was the co-depletion of Sec24a and 24b isoforms (*p*-value < 0.0001 ****). The co-depletion of Sec24c and 24d reached a *p*-value of 0.0004 (***). Significance for the single isoform depletions are as follows: Sec24a, 0.0192, (*); Sec24b, 0.0020 (**); Sec24c, ns; Sec24d, 0.0018 (**). Panel (**E**) shows the cell cycle distribution of these populations at the time of the collection of the GFP flow cytometric data.

To determine if COPII transport is functionally involved in HPV16 infection, we used siRNA to deplete HeLa cells of various components participating in this pathway. We monitored infection by flow cytometry using an HPV16 PsV containing a pseudogenome encoding GFP. Sar1 is the small GTPase that regulates the formation or assembly of COPII vesicles involved in the ER export of proteins [45]. We used siRNA to reduce the expression of both Sar1a and Sar1b as the two paralogs may function in unison at the same ERES site to drive vesicle generation [46]. With the combined depletion, we were able to reduce HPV16 PsV infection by approximately 55%, as shown in Figure 3B. This figure represents a composite of nine separate experiments, which reached a significance of <0.0001. A representative Western blot demonstrating the loss of both paralogs is shown in the upper panels of Figure 3A.

We also targeted Sec24, a structural component of the inner COPII cage, which also participates in cargo selection. There are four different isoforms of Sec24 in mammalian cells. These isoforms exhibit cargo-specific variability in importance [47]. We found a minimal effect on infection when we singly depleted the four isoforms: Sec24a (*p*-value 0.0192), 24b (*p*-value 0.0020), 24c (*p*-value not significant), or 24d (*p*-value 0.0018) (Figure 3D), consistent with described functional redundancy [47]. However, when we depleted selected combinations of the isoforms, we found that depletion of both Sec24a and 24b decreased pseudovirus infection. We found an average of approximately 60% reduction in infection over six separate experiments (*p*-value < 0.0001). There was about a 20% infection reduction with the combined depletion of Sec24c and Sec24d isoforms (*p*-value 0.0004). The pairing of Sec24a/b and Sec24c/d into two classes with a tendency toward distinct cargo selectivity has been previously described [48]. The corresponding representative Western blots are shown in the upper panel of Figure 3C.

The COPII outer coat lattice is composed of Sec13 and Sec31. We could consistently achieve a partial reduction in PsV infection with Sec13 depletion, achieving approximately 40% reduction in infection over nine experiments (Figure 3B). This partial decrease was reproducible and reached significance (*p*-value < 0.0001). However, we were unable to reproducibly deplete Sec31. We observed a robust reduction in infection when knockdown was achieved, but we were not able to replicate this knockdown reliably enough to form a definitive conclusion about the role of Sec31 in HPV16 PsV infection.

We also examined the effect of depletion of TANGO1. TANGO1, an ERES-localized transmembrane protein can act as an ERES organizer and a cargo receptor [48,49,50,51]. It also serves to recruit ERGIC membranes to the ERES in order to generate the extended vesicle sizes required for the secretion of collagen and other large cargo [30]. TANGO1 does not accompany the resultant vesicles distally from the ERES [49]. As we observed PsV particles in extended vesicles (shown in Figure 1), reminiscent of the ones generated for procollagen export, we considered that the involvement of this mechanism was possible. As shown in Figure 3, depletion of TANGO1 did negatively affect PsV infection. We found approximately 60% reduction in infection over 12 experiments (Figure 3B) with a significance of <0.0001. The accompanying Western blot is shown in Figure 3B.

Additionally, we confirmed that the reduction in these proteins did not adversely affect cell cycle progression. The cell cycle profiles from a representative depletion experiment are shown in Figure 3E. The distribution among cell cycle stages was not affected by these various depletions indicating that the observed effects on infection were not simply attributable to cell cycle arrest.

### 3.4. Depletion of COPII Proteins Affects PsV Trafficking

**Figure 4:**
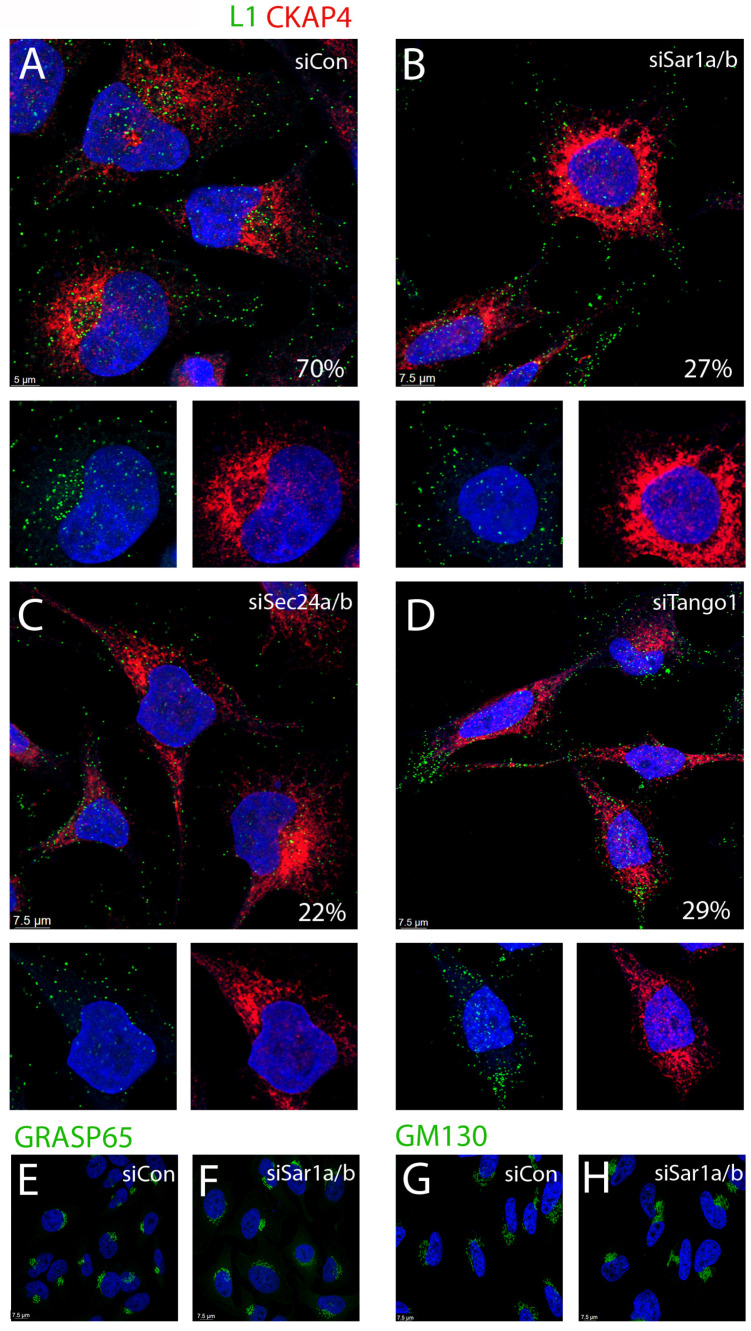
Perinuclear distribution of PsV under conditions of COPII silencing. At 48 h post siRNA transfection, cells were trypsinized and replated onto glass coverslips in a 24-well vessel. The perinuclear distribution of L1, relative to the ER sheet protein, CKAP4, was determined under each condition as noted in the panels (**A**–**D**). An enlarged split channel image of the indicated area is shown below each larger panel. The percentage of cells (from >50 cells) that showed a distinct perinuclear distribution of L1 is shown in each panel. In each instance, L1 is displayed in the green channel and CKAP4 is in the red channel. Nuclei are stained with DAPI (blue channel) in all panels. The effect of Sar1a/b silencing on the distribution of two Golgi markers is shown in panels (**E**–**H**) as indicated. The percentage of L1 staining determined to be perinuclear is indicated in each panel.

**Figure 5:**
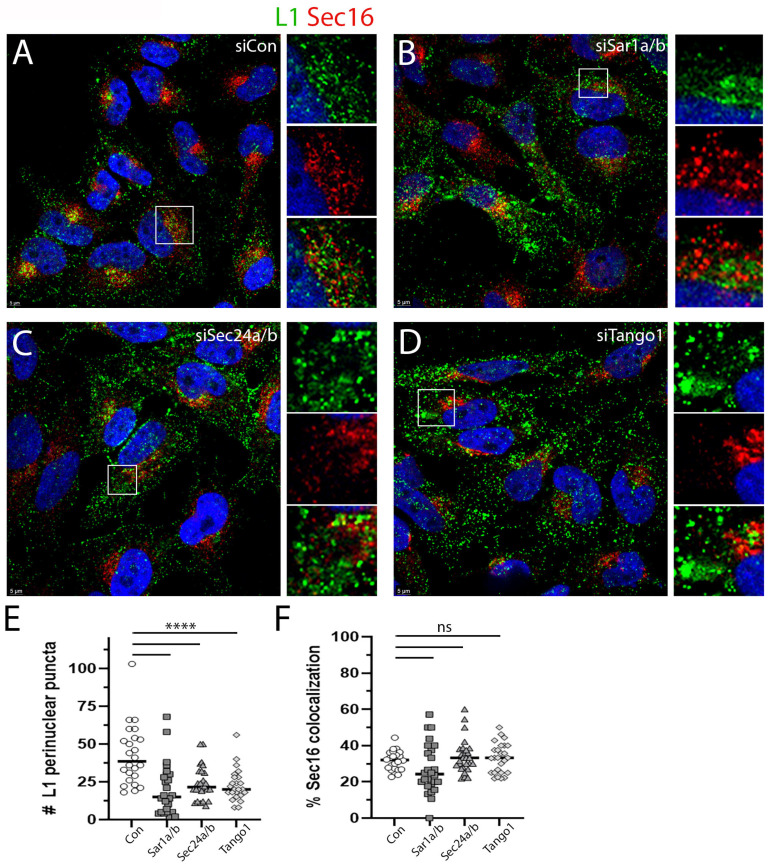
Colocalization of PsV with Sec16 following COPII silencing. The localization of L1 to the ERES was determined with co-staining of L1, and the ERES marker protein, Sec16, was determined under each condition as noted in panels (**A**–**D**). An enlarged split channel image of the indicated area is shown below each larger panel. In each instance, L1 is displayed in the green channel and Sec16 is in the red channel. Nuclei are stained with DAPI (blue channel) in all panels. The number of perinuclear puncta from central Z-stack sections of 24–28 cells was determined for each condition as shown in panel (**E**). Significance of <0.0001 is indicated by ****. The colocalization of L1 with Sec16 within these perinuclear puncta was also determined and is shown in panel (**F**). Non-significance is designated ns.

We examined the localization of capsids endocytosed under the conditions of COPII depletion. We did not include Sec13a in this analysis as the observed 40% decrease in infection would not allow us to unequivocally attribute an L1 localization phenotype to this loss of expression. As HPV16 capsids traffic asynchronously, these analyses can be difficult to interpret, and quantification of colocalization must be approached cautiously. We considered this issue would be especially problematic for the transient passage through the ER. Therefore, we decided to evaluate both the perinuclear character of the HPV16 PsV capsids relative to a marker of the ER sheet, CKAP4 (shown in Figure 4), and the colocalization of L1 with the ERES marker, Sec16 (Figure 5). For all panels, we selected a Z stack slice, which showed the strongest signal for the marker protein analyzed.

HeLa cells that had been transfected with an siRNA control showed the expected distribution of capsids. At 24 h post infection, we found approximately 70% of cells with distinct perinuclear capsid distribution (green channel) defined by CKAP4 localization (red channel). This was determined by examination of Z stack images of 50 infected cells. A representative image is shown in Figure 4A. As expected, L1 staining was also clearly detectable in vesicles throughout the cytoplasm. An enlarged region, with split color channels, is shown in the lower panels to allow better appreciation of the well-described perinuclear PsV. However, the cells in which COPII transport had been disrupted showed a markedly different distribution of L1. This is shown following depletion of Sar1a/b in Figure 4B. Under this condition we found that L1 exhibited a distinct perinuclear pattern in only 27% of the cells. The majority of the L1 staining was diffusely localized. Depletion of Sec24a/b reduced the perinuclear distribution to 22% (Figure 4C). TANGO1 depletion resulted in only 29% of infected cells with a perinuclear L1 distribution (Figure 4D). The distribution of CKAP4 does not noticeably shift under these conditions of COPII silencing. We also examined the effect of Sar1a/b depletion on the distribution of two Golgi markers, GRASP65 and GM130. As shown in Figure 4E–H, neither marker is dramatically altered by disruption of COPII-mediated transport.

The observed L1 distribution pattern under COPII depletion conditions shown in Figure 4 did not resemble an ERES pattern, but we wanted to determine if these peripheral vesicles contained ERES markers. This staining is shown in Figure 5, with anti-L1 staining shown in the green channel and anti-Sec16 shown in the red channel. As expected, the siRNA transfected control cells showed good perinuclear distribution of L1 with a subset that colocalized with Sec16 (Figure 5A). A split channel image is shown below. As shown in Figure 4, the distribution of L1 under all the COPII-depletion conditions is dramatically altered. The depletion of Sar1a/b is shown in Figure 5B. The depletion of Sec24 a/b is shown in Figure 5C,D, illustrating an example of TANGO1 depletion. In each instance, a magnified and split channel image is shown in an adjacent panel. Interestingly, we found significantly fewer perinuclear L1 puncta under all depletion conditions as quantified in Figure 5E, but the colocalization of L1 and Sec16 within the remaining perinuclear population was unchanged across the conditions (Figure 5F). The more peripheral staining colocalizes minimally with Sec16. As the silencing did not completely ablate infection as shown in Figure 3, this phenotypic subset is unsurprising. These data further illustrate the aberrant PsV trafficking phenotype under conditions of COPII inactivation and also demonstrates that this pattern does not represent distal ERES elements. A priori, we would have anticipated the retention of PsV within the ER/ERES as a depletion of COPII elements should prevent their exodus. However, this locale is also occupied by the omegasome, a site of phagophore generation [50,51].

**Figure 6:**
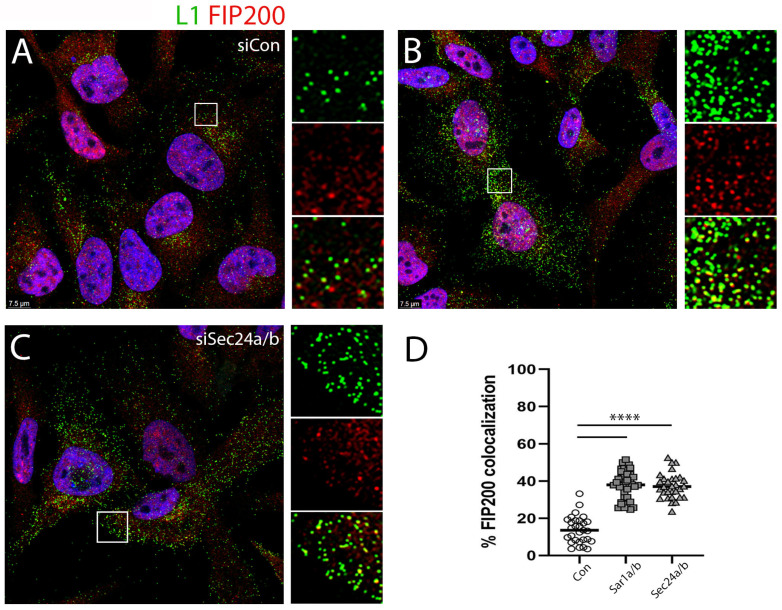
Colocalization of PsV with FIP200 following COPII silencing. The colocalization of L1, detected with FIP200, was determined following transfection with the siRNAs as indicated for each panel (**A**–**C**), as indicated. The staining for L1 is shown in the green channel, and the staining for FIP200 is shown in the red channel. Nuclei are stained with DAPI (blue channel) in all panels. Panel (**D**) shows the quantification of colocalized L1 and FIP200 in cellular regions distal to the nucleus. Significance of <0.0001 is indicated by ****.

There exists a well-described interplay between anterograde COPII vesicle generation and autophagosome biogenesis [51]. Omegasome formation requires both the UNC-51-like kinase (ULK) complex and its scaffold protein FIP200 (also known as RB1CC1) [52,53]. FIP200 localizes to the isolation membrane, and recent studies have suggested that under conditions of cell stress, a remodeling of the endomembrane system occurs, which results in an enrichment of FIP200 in the ERES/ERGIC region [54]. Therefore, we wondered if, in the absence of COPII trafficking, the PsV was being shunted into this physically opposed pathway. We found that a subset of perinuclear L1 colocalized with FIP200 even under normal infection conditions (Figure 6A). This is unsurprising given the described localization of FIP200 to ERES membranes [55]. The distal L1-containing vesicles under this condition are largely free of FIP200 staining (Figure 6D). Most importantly the peripheral PsV staining found under conditions of COPII interference was found to be largely coincident with FIP200 staining. This is shown in Figure 6B,C for siRNA depletion of Sar1a/b and Sec24a/b, respectively. The degree of L1 and FIP200 colocalization in peripheral vesicles is quantified in Figure 6D. This quantification was determined across 30–40 distal regions of 23–32 cells/condition.

### 3.5. Co-Immunoprecipitation of COPII Cargo Acceptor with HPV Capsids

**Figure 7:**
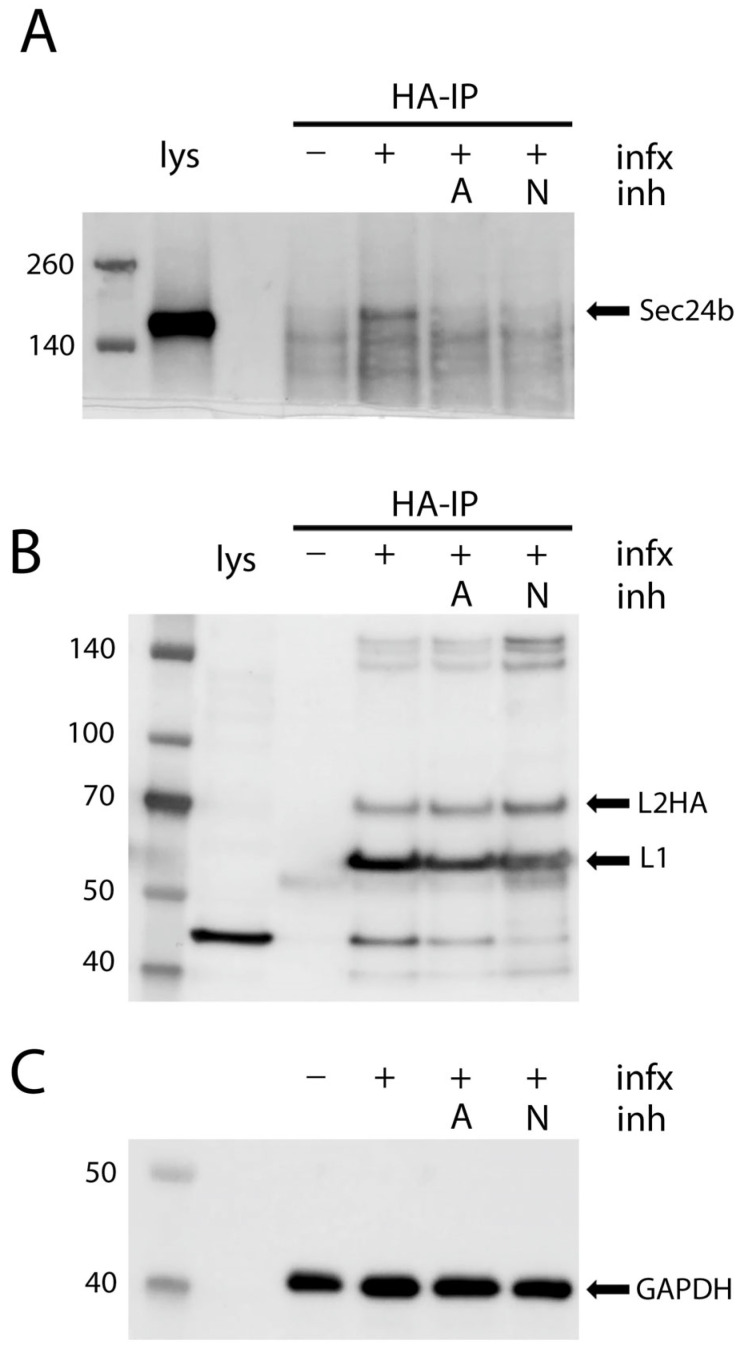
Isolation of Sec24b with HPV16 PsV. HeLa cells were infected with HPV16 PsV composed of L1 and an L2 containing five carboxyl terminal HA epitope tags (p16Ll2-laugh). At 6 h post infection some cells were treated with aphidicolin to a final concentration of 5 μg/mL. Another group was treated with NH_4_Cl to a final concentration of 20 mM and a third group was left untreated. Infection was allowed to continue for an additional 18 h. At this point the cells were crosslinked with DSP as detailed in the Materials and Methods. Protein complexes were immunoprecipitated from cell lysates with an anti-HA reagent and protein content determined by Western blotting. Panel (**A**) shows the detection of Sec24b of the various treatment conditions as indicated, +/− infection as denoted with “infx”. Lys denotes cell lysate included as a protein migration/detection control for Sec24b, indicated with an arrow. Inhibitor-treated samples are indicated with “inh”, while aphidicolin-treated samples are indicated by “A” and NH_4_Cl-treated samples indicated by “N”. The retained association of L1 with L2-HA is demonstrated in panel (**B**). Lane 1 shows HeLa cell lysate that was not subjected to immuno-isolation. The well-described lower molecular cross-reactive cellular protein is evident. The migration of L2-HA and L1 are indicated by arrows. GAPDH detection of the cell extracts used for the immunoprecipitation are shown in panel (**C**).

We wished to biochemically demonstrate an association between HPV16 capsids and COPII vesicle components. The confounding issue for this experiment is the slow entry of HPV16 and the lack of synchrony during intracellular trafficking. To maximize sensitivity in the detection of associated proteins in these experiments, we employed a PsV containing an L2 with multiple carboxyl terminal HA tags and performed co-immunoprecipitation experiments following mild crosslinking. This PsV shows no loss of infectious titer. We used an anti-HA antibody followed by Protein G coupled magnetic beads to isolate PsV and associated proteins. Figure 7A shows immunoprecipitation of the COPII cargo binding protein Sec24b with L2-HA. The HA antibody did not nonspecifically isolate Sec24b from uninfected cells. Immunoprecipitation from infected cells that were treated with the infection inhibitors aphidicolin and NH_4_Cl was unsuccessful indicating complex formation only under conditions which would allow for post-Golgi trafficking. Aphidicolin has been shown to prevent HPV16 infection by preventing their egress from the Golgi [16,17]. NH_4_Cl blocks the transit of PsV through the endosomal compartment prior to arrival in the Golgi [2,56]. We could only consistently isolate Sec24b in association with L2-HA in the cells that had unimpeded infection. We were unable to isolate Sec24a in this complex.

The coprecipitation of L1 with the HA-tagged L2 under these conditions is shown in Figure 7B. Immuno-isolation of L2 resulted in the co-isolation of L1 in all instances. This confirms continued association of the two capsid proteins throughout cellular trafficking as previoulsy described [14,24]. This analysis demonstrates a physical interaction, albeit to a low degree, between HPV16 PsV and Sec24b, further supporting the transit of the virus through the COPII compartment.

## 4. Discussion

Intracellular vesicular trafficking is a complicated system that maintains balance among membrane-bound organelles and their cargoes. Vesicle formation may occur at various membrane sites and different effector and coat proteins mediate these distinct events, but the general character of these processes is shared. In each instance, bud initiation is followed by cargo selection, coat assembly around the nascent bud which is followed by vesicle scission. To remain within the scope of this study, we will only summarize COPI- and COPII-coated vesicle transport.

**Figure 8:**
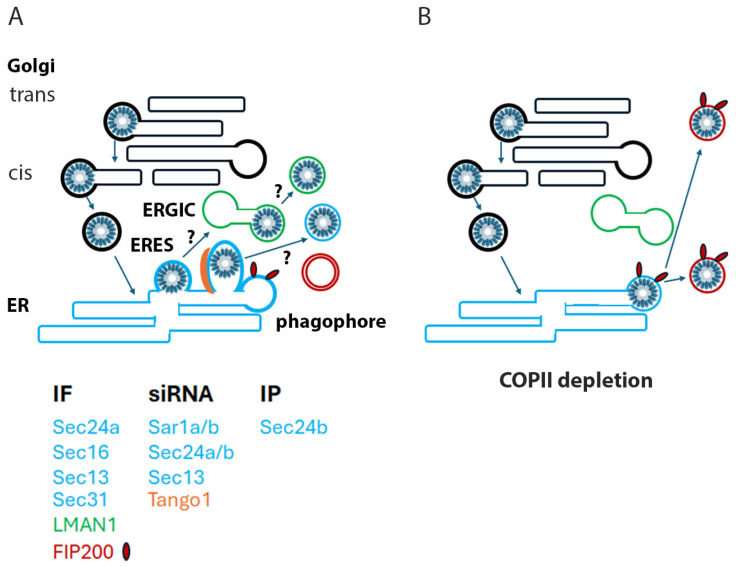
Experimental model. Panel (**A**). This model illustrates our concept of HPV16 PsV trafficking in interphase cells. PsV follows the well-established retrograde route through the Golgi stacks (black) into the ER utilizing COPI carriers (heavy black). Within the ER (blue), PsV were found associated with multiple COPII proteins within the ERES (heavy blue), some of which were demonstrated to assist infection via siRNA knockdown experiments. PsV also colocalized with ERGIC markers (green), but as Tango1 (orange) may induce the relocalization of the ERGIC membrane to the ERES, this is not definitive proof of ERGIC transit. Therefore, PsV-containing vesicles could be generated from the ERES or the ERGIC. These vesicles move towards the condensed chromatin following entry into mitosis. FIP200 also localizes to the ERES and is involved in phagophore generation (red). In the absence of COPII function, the PsV is shunted into FIP200-associated vesicles as illustrated in panel (**B**). The table below lists the described participating proteins, and the type of evidence provided. The proteins are color-coded based on their subcellular localization. COPI vesicles budding from the Golgi are indicated with heavy black outlines, and COPII vesicles emerging from the ERES have a heavy blue outline.

ER to Golgi transport is mediated by COPII-coated vesicles (Figure 8) (reviewed in [57]). Vesicle formation is initiated by the cytosolic GTPase Sar1 which upon activation recruits the inner coat proteins Sec23 and Sec24. Sec 23 promotes Sar1 GTPase activity and Sec24 directly interacts with cargo and adaptor proteins to mediate cargo loading [57,58,59,60,61]. The outer shell of the coated vesicle is composed of a heterodimeric lattice of Sec13 and Sec31. Vesicle biogenesis occurs at the ERES, discrete sites on the ER membrane, which can be defined by the presence of Sec16, a COPII assembly scaffold protein [27,36,58,59,60,61]. Typically, mammalian COPII vesicles traffic to the proximal ERGIC, and their cargo is subsequently delivered to the Golgi via COPI-coated vesicles [62]. Irregular and bulky cargo require a modification of the typical COPII generation process. TANGO1, an ERES resident protein, generates extended vesicles via the relocalization of ERGIC membranes to the nascent bud [30]. Fusion of the delivered membrane is enabled by SNARE (soluble N-ethylmaleimide sensitive factor attachment protein receptors) proteins including syntaxin 18 (STX18) [63]. TANGO1 also serves as a sorting receptor for a subset of these large cargos [49,64,65]. However, the distribution of different cargo and the dependence on specific receptors is not solely driven by cargo size, as unlike the secretion of procollagens I, III and VII, procollagen IV secretion is not dependent upon these auxiliary components [66,67,68,69].

COPI-coated vesicles promote both the retrograde transport of cargo proteins from the trans-Golgi through the Golgi stacks ending with delivery to the ER and also the anterograde passage between the ERGIC and the ER (reviewed in [44]). The formation of these vesicles is dependent on the Arf1 GTPase and the assembly of the heptameric coatomer protein complex, which interacts with the specific cargo. It was recently demonstrated that HPV transit through the Golgi is dependent upon a functional COPI pathway [12]. The L2 capsid protein protrudes from the trafficking vesicle to mediate contact with cytoplasmic host proteins. There appear to be a sequential relay of L2-interacting proteins that chaperone the virion from early endosomes to the Golgi, the most well defined being the retromer complex (reviewed in [70]). The finding that L2 interacts with the COPI complex cleanly delineates another step in the pathway to the nucleus. HPV virions accumulated in both the trans and cis Golgi regions when COPI trafficking was disrupted. As suggested by these authors, this points to a possible role of COPI in mediating the delivery of HPV from the Golgi to the ER. However, they did not identify COPI vesicles as the HPV capsid-containing vesicles on the mitotic chromosomes, nor do they resemble them morphologically. In accord with this, we could not colocalize the β-coatomer with L1 on mitotic chromosomes, although colocalization within other vesicles was clearly observed.

Our current work extends from our previous observation [23], wherein also shown in Figure 1, the HPV-containing vesicles are morphologically reminiscent of the extended tubular vesicles, ranging from 60 nm vesicles to 200 nm tubules, generated by the COPII anterograde transport pathway to mediate the export of bulky and irregular cargo [27,28,29]. We also found visual evidence of 50 nm particles within the ER and budding from the ER. Extending from this TEM data, using a monoclonal antibody against HPV16 L1, we demonstrated that PsV colocalize with Sec16, an ERES resident protein (Figure 2A). Although HPV L2 has been previously colocalized with ER sheet proteins [19,20], the localization of PV proteins with secretory regions of the ER has not been reported. We describe the colocalization of L1 with both ERES, COPII vesicle components and an ERGIC marker. The outer coat proteins Sec13a and Sec 31a and the inner coat protein, Sec24a, were all found in association with L1 (Figure 2B–E). Despite this we were unable to colocalize the coat components with L1 within the central condensed regions of mitotic chromosomes, although there was clear localization of L1 adjacent to the chromosomes in prophase cells (Figure 2F). It is possible that the chromatin sterically occludes antibody access to the coat lattice, but this is unlikely to fully explain this observation. The generally accepted dogma is that coat proteins remain associated with the vesicle until fusion with a recipient membrane. However, recent work has challenged this precept [71] and suggests that the COPII complex defines the boundary between the ER and the ERES. In this model, the COPII assemblage is essential for cargo selection and ER export but through a coated membrane collar rather than via mobile vesicles. This idea proposes the cooperation of COPI in anterograde traffic at the ERES membrane not at the later ERGIC location. In this scenario, the COPII coat would not accompany the PsV cargo beyond the ERES-generated tubular extensions. As COPI mediates the retrograde transit of PsV through the Golgi, it would not be possible to dissect an additional role at the subsequent stage of PsV transit towards the nucleus.

In this study, we have demonstrated a functional role of an intact COPII pathway at a late step in HPV PsV trafficking. Silencing of the Sar1a/b GTPase and both inner and outer coat proteins reduced PsV infection. However, infection was not completely blocked despite substantial decreases in protein expression levels. Perhaps relatively few PsV are released into the ER/ERES so minimal operational COPII complexes are required to escort them. Alternatively, as previously suggested, COPI can inefficiently coordinate anterograde trafficking in the absence of a functioning COPII pathway [72]. Another possibility is that there are multiple mechanisms of egress from the Golgi that can lead to infection. We cannot currently distinguish among these possible explanations.

We also showed that the transport accessory protein TANGO1 participates in this transport. The ER SNARE protein STX18 acts in concert with TANGO1 for the secretion of some bulky cargoes [69]. Intriguingly, previous studies have implicated STX18 in PV entry and infection [8,21]. Engineered PsV that are unable to interact with STX18 showed unimpaired localization to the Golgi and subsequently to the ER. However, these mutant PsV were not delivered to the nucleus, consistent with a role for STX18 in ER egress.

In accordance with these data supporting a role for COPII-mediated transport in HPV16 PsV infection, we were able to isolate Sec24b with HPV16 PsV during entry. We immunoprecipitated PsV using an HA epitope tagged L2. This co-immunoprecipitation, albeit weak, only occurred under conditions in which the PsV was allowed to traverse beyond the Golgi. Preventing passage into either the Golgi, with aphidicolin, or the late endosomes, with NH_4_Cl, prevented the coprecipitation of Sec24b. L1 was associated with L2 under all conditions, consistent with previous reports demonstrating the accompaniment of L1 with L2 in late trafficking [13,23].

Due to the asynchrony of HPV infection, which results in particle distribution throughout the trafficking pathway even at 24 h post infection, it is not possible to achieve complete colocalization of viral proteins with any compartment. It is known that arrest of the cell cycle in G1 causes an enrichment of the PsV in the Golgi. In early prophase, the secretory compartment is dramatically reorganized [73], and COPI-mediated budding from the Golgi is escalated [74]. Possibly, entry into mitosis would result in transit of a cohort of PsV from the Golgi to the ER and ERES and, subsequently, to the condensing chromatin upon nuclear envelope dissolution. This idea is supported by the observation that L2 loses Golgi colocalization during early prophase [21]. However, we were unable to increase PsV concentration in the ERES following aphidicolin release (unpublished data).

These data represent an interesting convergence with the observation that there is an inverse relationship between successful HPV16 PsV infection and activation of autophagy [75,76]. Omegasomes, the structural platforms for autophagosome biogenesis, have long been recognized as being associated with ER, and multiple studies have implicated the ERES as a site of their nucleation and expansion [77,78]. It is interesting to consider that HPV16 PsV trafficking is balanced between these two physically coinciding but mechanistically opposed pathways. The colocalization of FIP200, described as a scaffold protein on autophagic vesicles, with L1 on the peripheral vesicles induced by COPII depletion, belies this confluence. It will be important to confirm this possibility with future experimentation. We summarize these ideas in the model shown in Figure 8.

## Figures and Tables

**Table 1:** siRNA sequences and suppliers.

Sar1a	#1 GAACAGAUGCAAUCAGUGA, #2 CCAGUAUAUUGACUGAUGU (Sigma)
Sar1b	#1 GCAUAACUUGAAUUCAAUA, #2 CUACCUUCCUGCUAUCAAU (Sigma)
Sec13	#1 CAUGUGAGCUGGUCCAUCA, #2 GGUCGUGUGUUCAUUUGGA, #3 CCAUCUCCCUGCUGACUUA, #4 GUAAUUAACACUGUGGAUA (Dharmacon)
Sec24a	#1 GGACGUACAUCAAUCCUUU, #2 CCAAGAAGGUAUUACAUCA, #3 GUGGUUACCUCCAGUACAA, #4 CAGUAGUUACGACGAGAUU (Dharmacon)
Sec24b	#1 CUUCAGAGACCUAACGCAA, #2 CCAGAUUCAUUUCGGUGUA, #3 CUUCAUUGAUCAACGUAGA, #4 GCUAUAGAGUAAACGAUGU (Dharmacon)
Sec24c	#1 GCACAGAGAUCCCGGUACA, #2 UGGCUGAUCUAUAUCGAAA, #3 CCUUUCAGGUGGAGAACGA, #4 CCUGGAUCAUACCGGCAAA (Dharmacon)
Sec24d	#1 GGUAAAUCACGGCGAGAGU, #2 GAUCUCAACUGAUGAACGA, #3 UUGAAGGUCAUCCGGGAAA, #4 CGUUAGAUGUCAAGAGUAC (Dharmacon)
TANGO1	GAUAAGGUCUUCCGUGCUU (Sigma)
Universal negative control	predesigned (Sigma #SIC001)

**Table 2:** Antibodies.

Target (Name)	Usage (Dilution)	Source (Catalog #)
CKAP4	IF (1/800)	Proteintech (16686-1-AP)
GAPDH	WB (1/1000)	Novus (NBP2-27103)
HA Tag	IP (4 μg/sample)	Epicypher (13-2010)
HA Tag	WB (1/100)	Santa Cruz Biotechnology (F-7)
HPV16 L1 (H16.7E)	IF (1/300)	Neil Christensen
HPV16 L1 (Camvir-1)	WB (1/10,000)	Abcam (ab69)
FIP200	IF (1/400)	Abcam (ab227726)
GM130	IF (1/200)	BD Biosciences (610822)
GRASP65	IF (1/500)	Invitrogen (PA3-910)
LMAN1	IF (1/200)	Novus (NBP3-04910)
Sar1A	WB 1/1000	Novus (NBP2-20261)
Sar1B	WB 1/1000	Novus (NBP1-32725)
Sec13	WB (1/1000)	Invitrogen (PA5-21339)
Sec16A	IF (1/300)	Invitrogen (PA5-52182)
Sec24A	IF (1/500)	Proteintech (15958-1-AP)
WB (1/2000)		
Sec24B	WB (1/1000)	Cell Signaling Tech (12042)
Sec24C	WB (1/1000)	Cell Signaling Tech (14676)
Sec24D	WB (1/1000)	Cell Signaling Tech (14687)
Sec31A	IF (1/300)	Abcam (ab86600)
WB 1/1000		
TANGO1/MIA3	WB (1/1000)	Abcam (ab244506)
Donkey anti-mouse IgG Alexa Fluor 488		
	IF (1/1000)	Invitrogen (A32766)
Donkey anti-rabbit IgG Alexa Fluor 594		
	IF (1/1000)	Invitrogen (A21207)
Goat anti-mouse IgG HRP		
	WB (1/5000)	Invitrogen (A16072)
Goat anti-rabbit IgG HRP		
	WB (1/5000)	Invitrogen (A16110)

## Data Availability

All data, methods, and reagents generated will be freely available.

## Abbreviations

The following abbreviations are used in this manuscript:

COPI	coat protein complex I
COPII	coat protein complex II
ER	Endoplasmic reticulum
ERES	ER exit sites
ERGIC	ER-Golgi intermediate compartment
HPV	Human papillomavirus
PsV	Pseudovirus
STX18	Sorting nexin 18
TANGO1	transport and Golgi organization 1 protein
TEM	transmission electron microscopy
